# Selective Anti‐Inflammatory Actions of Curcumin on Cytokine Pathways in Major Depressive Disorder

**DOI:** 10.1002/brb3.71672

**Published:** 2026-08-12

**Authors:** Tangcong Chen, Yueyang Luo, Mengqi Niu, Jing Li, Mengdie Li, Yingqian Zhang, Michael Maes

**Affiliations:** ^1^ Sichuan Provincial Center for Mental Health Sichuan Provincial People's Hospital School of Medicine University of Electronic Science and Technology of China Chengdu China; ^2^ Key Laboratory of Psychosomatic Medicine Chinese Academy of Medical Sciences Chengdu China; ^3^ Department of Psychiatry Medical University of Plovdiv Plovdiv Bulgaria; ^4^ Research Institute Medical University Plovdiv Plovdiv Bulgaria; ^5^ Research and Innovation Program for the Development of MU‐PLOVDIV (SRIPD‐MUP) Medical University of Plovdiv Creation of a Network of Research Higher Schools National Plan for Recovery and Sustainability European Union–NextGenerationEU Plovdiv Bulgaria; ^6^ Kyung Hee University Seoul Republic of Korea

**Keywords:** curcumin, cytokines, inflammation, major depressive disorder

## Abstract

**Background:**

Major depressive disorder (MDD) is a prevalent mental illness with a significant disease burden. It is characterized by immune‐inflammatory dysregulation.

**Methods:**

This ex vivo study assessed curcumin's immunomodulatory effects in 18 MDD patients and 18 healthy controls (HC). Whole blood samples underwent LPS‐ and PHA‐stimulation to evaluate immune sensitization with curcumin treatment at concentrations of 2, 20, and 200 ng/mL.

**Results:**

MDD patients exhibited marked activation of the immune‐inflammatory response system (IRS), M1 macrophages, T helper (Th) 1, growth factors, and chemokine profiles, whereas M2, Th2, and the compensatory immunoregulatory system (CIRS) were less activated, confirming IRS sensitization in MDD. Partial regression analysis confirmed positive correlations between immune biomarkers (e.g., IL‐1β) and depression severity scores. In patients with MDD, curcumin selectively and significantly downregulated the overactivated IRS, M1, Th1, and chemokine profiles. Notably, curcumin significantly decreased pro‐inflammatory chemokines, such as CCL11, CXCL10, and CCL27, and exhibited inhibitory effects on key inflammatory factors, including TNF‐α. Although curcumin did not fully normalize the IRS profiles or significantly affect M2, Th2, or CIRS profiles, its specific inhibition of core inflammatory pathways clearly demonstrates its ability to selectively modulate aberrant immune responses in MDD. Paradoxically, in HC, curcumin showed mild immune‐stimulating effects.

**Conclusions:**

These findings demonstrate that curcumin exerts selective anti‐inflammatory effects by effectively targeting and suppressing overactivated core inflammatory pathways (e.g., IRS/M1/Th1 and related chemokines) in MDD patients, thereby partially restoring immune homeostasis. This supports the potential role of curcumin as an adjunctive therapeutic strategy for MDD. However, as these findings are derived from an ex vivo model, further in vivo studies are required before clinical recommendations can be made.

## Graphical abstract

Curcumin partially attenuates IRS/M1/Th1 immune activation in MDD patients ex vivo, but fails to normalize it. Paradoxically, it upregulates immune profiles in healthy controls. Its immunomodulatory effects are state‐dependent, suggesting cautious use in non‐depressed individuals.

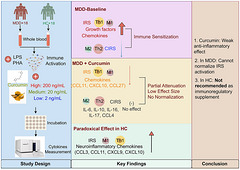

**Abbreviations**
ANOVA: analysis of variance
BIA: bioelectrical impedance analysis
BMI: body mass index
CIRS: compensatory immunoregulatory system
FDR: false discovery rate
FFS: FibroFatigue scale
FI: fluorescence intensities
GEE: generalized estimating equation
GLM: general linear model
HAMA: Hamilton Anxiety Rating Scale
HAMD: Hamilton Depression Rating Scale
HC: healthy controls
ICH‐GCP: International Conference on Harmonization in Good Clinical Practice
IFN‐γ: interferon‐γ
IL: interleukin
IRS: immune‐inflammatory response system
LPS: lipopolysaccharide
MDD: major depressive disorder
MINI: Mini International Neuropsychiatric Interview
NAIOSs: natural anti‐inflammatory and anti‐oxidative substances
PHA: phytohemagglutinin
Th1: T helper 1
TNF: tumor necrosis factor
TRD: treatment‐resistant depression

## 1 Introduction

Major depressive disorder (MDD) is a prevalent mental health disorder characterized by emotional and cognitive symptoms, such as anhedonia, feelings of guilt, and suicidal ideation, and physiosomatic symptoms like fatigue, weight loss, and appetite changes, as well as insomnia (GBD 2017 Disease and Injury Incidence and Prevalence Collaborators 2018). MDD affects around 300 million people globally, ranked third in disease burden in 2018, and is projected to become the leading cause by 2030 (Malhi and Mann 2018; Nagy et al. 2020). Developing effective strategies for precise diagnosis and therapeutic intervention of depression remains one of the most pressing challenges in contemporary medical practice.

Although the pathogenesis of MDD is not yet clear, many studies have reported a profound association between MDD and immune system dysfunction (Maes et al. 2025). Studies have shown that the excessive secretion of immune signaling molecules, especially pro‐inflammatory cytokines, may play a crucial role in the onset and maintenance of the acute phase of severe MDD (Maes et al. 1995). Subsequent meta‐analyses studies have indicated that in patients with MDD, the levels of interleukin‐6 (IL‐6), interferon‐γ (IFN‐γ), and other cytokines, especially tumor necrosis factor (TNF) indicators, are elevated, indicating the activation of the immune‐inflammatory response system (IRS) (Dowlati et al. 2010). A meta‐analysis indicates that patients with MDD exhibit abnormal levels of various cytokines in peripheral blood, such as elevated IL‐6 and TNF‐α, alongside reduced IFN‐γ, with significant heterogeneity observed across studies (Köhler et al. 2017). Antidepressant treatment significantly reduces levels of peripheral inflammatory markers like IL‐6 and TNF‐α, but no direct association has been found between these changes and treatment response (Köhler et al. 2018). Accumulating evidence suggests that M1 macrophages, T helper 1 (Th1) cells, and T helper 17 (Th17) cells exhibit elevated activity in MDD patients, collectively driving neuroinflammation and contributing to core depressive symptoms (Almulla and Maes 2025). Almulla et al. (2024) reported that patients with MDD exhibited increased levels of M1 macrophages, Th1 cells, and pro‐inflammatory cytokines such as IL‐6 and TNF‐α, which are strongly associated with M1 macrophage activation. In addition, increased concentrations of IL‐16 and other cytokines related to Th1 cell activation were found. However, the role of Th17 cells in MDD remains relatively complex, with some studies suggesting potential associations between Th17 cell activation and the severity of MDD. These immune alterations may contribute to the pathophysiology of MDD by promoting neuroinflammation and impairments in synaptic plasticity and neurotransmitter function.

There is now evidence that an imbalance in activation of the IRS versus the compensatory immunoregulatory system (CIRS) plays a key role in MDD (Maes and Carvalho 2018). The CIRS is the component of the immune system responsible for maintaining immunological homeostasis through immunoregulatory effects, which include negative immunoregulatory cytokines like IL‐10 and IL‐4, and T regulatory cells (Maes and Carvalho 2018). The aforementioned authors developed the “IRS‐CIRS imbalance” hypothesis of MDD, positing that CIRS activities are comparatively diminished during the acute phase of MDD, hence facilitating net IRS activation (Debnath et al. 2021). Furthermore, Ahmetspahic et al. (2018) have found abnormalities in the adaptive immune system of MDD patients, including impaired maturation of Th2/Th17 cells and a reduction in regulatory B cells, which further supports the core role of immune regulation in the pathology of depression. It should be emphasized that the imbalance of IRS‐CIRS might be a biomarker for depression and may lead to the development of new therapies targeting immune pathways (Debnath et al. 2021). These studies provide an important basis for understanding the neuro‐immune mechanisms of depression and developing immune‐modulating therapies.

A prominent feature of MDD during its acute phase is sensitization of IRS (M1, Th1, and Th17) and CIRS (M2, Th2, and Treg) immune profiles (Debnath et al. 2021; Maes et al. 2024). This sensitization can be assessed experimentally using whole blood assays stimulated with immunogens like lipopolysaccharide (LPS) (mainly activating the innate immune system) and phytohemagglutinin (PHA) (mainly activating T cells); these assays measure exaggerated IRS versus CIRS cytokine production, reflecting hyperactive innate and adaptive immune pathways in the absence of overt infection (Maes et al. 1999b). Critically, such immune imbalances correlate strongly with the severity of affective and psychosomatic symptoms (Maes et al. 2022). This ex vivo model is frequently used to examine the effects of antidepressant drugs on IRS versus CIRS activities (Maes 2001; Maes et al. 1999a).

Many MDD patients experience no or small improvements after treatment with antidepressants, a condition referred to as treatment‐resistant depression (TRD) (Voineskos et al. 2020). There is some evidence that TRD is associated with IRS activation, including increased levels of IL‐6 and TNF‐α (Maes et al. 1999a). Consequently, novel therapeutic strategies targeting upstream immuno‐inflammatory pathways are urgently needed to augment the effects of antidepressants (Maes et al. 1999a). Many repurposed drugs, including cytokine antagonists and COX‐2 inhibitors, and natural anti‐inflammatory and anti‐oxidative substances (NAIOSs), including curcumin, have been used toward that purpose (Maes et al. 2012).

Curcumin, the primary bioactive polyphenol in turmeric (*Curcuma longa*), along with other curcuminoids (e.g., demethoxycurcumin, bisdemethoxycurcumin), is found in the plant's rhizomes. Studies have shown that curcumin has antioxidant, anti‐inflammatory, and neuroprotective properties (Sharifi‐Rad et al. 2020). Clinical trials have demonstrated that curcumin might have clinical efficacy as an augmentation strategy in patients with MDD and might reverse the progression of MDD (Yu et al. 2015). Nevertheless, there is no data on whether curcumin can normalize the IRS sensitization in MDD.

Therefore, in this study, we aim to investigate whether curcumin can effectively attenuate immune sensitization in the acute phase of MDD compared to healthy controls. Specifically, we hypothesize that curcumin treatment will selectively suppress overactivated IRS, M1, Th1, and Th17 profiles, thereby rebalancing the immune system without significantly enhancing CIRS, M2, Th2, or Treg profiles. The optimal outcome would be that curcumin, at therapeutic concentrations, effectively blocks core inflammatory pathways in MDD patients, alleviating immune dysregulation and providing a mechanistic basis for its use as a novel therapeutic agent for this disorder. By elucidating the impact of curcumin on immune markers in MDD, we aim to address the existing knowledge gap and offer insights into its potential as an adjunctive treatment.

## 2 Materials and Methods

### 2.1 Participants

In this research, we enrolled 36 subjects, namely 18 healthy volunteers and 18 outpatients with MDD (Table S1). The latter were recruited at the Sichuan Provincial Center for Mental Health, Sichuan Provincial People's Hospital, Chengdu, China. The participants included males and females aged 18–65 years. Age was included as a covariate in all statistical analyses to adjust for potential age‑related effects. They met the DSM‐5 diagnostic criteria for MDD, with scores exceeding 16 on the 17‐item Hamilton Depression Rating Scale (HAMD‐17) (Hamilton 1960). Healthy volunteers, matched for age and gender to the patient group, were recruited from the healthy population in Chengdu through verbal communication. Exclusion criteria for MDD patients were other neuropsychiatric disorders, such as bipolar disorder, schizophrenia, schizoaffective disorder, organic mental disorders, substance use disorders, and autism spectrum disorders. Exclusion criteria for controls comprised any depression phenotype, including dysthymia (current and lifetime), as defined by DSM‐5.

Exclusion criteria for patients and controls were (a) neurological diseases (e.g., stroke, epilepsy, brain tumors, Parkinson's disease, Alzheimer's disease, and multiple sclerosis); (b) major medical diseases (e.g., autoimmune diseases, psoriasis, systemic lupus erythematosus, inflammatory bowel disease, rheumatoid arthritis, type 1 diabetes, chronic obstructive pulmonary disease, and cancer); (c) pregnant and breastfeeding women; (d) individuals with severe allergic reactions in the past month; (e) individuals with a history of infection in the past 3 months; (f) individuals who took therapeutic doses of antioxidants or omega‐3 supplements in the 3 months before the research; (g) individuals with a history of surgery in the past 3 months; and (h) individuals who frequently use analgesics.

All participants provided written informed consent before participating in the research. The study was conducted following international and Chinese ethical and privacy laws and received approval from the Ethics Review Committee of the University of Electronic Science and Technology of China (No. 30005).

### 2.2 Clinical Measurements

Trained researchers conducted semi‐structured interviews. A research psychologist familiar with all the scale instructions and items used the HAMD‐17 to assess the severity of depressive symptoms (Hamilton 1960; Zimmerman et al. 2013). The Mini International Neuropsychiatric Interview (MINI) was employed to make the diagnosis of MDD and rule out other Axis I psychiatric disorders (Pettersson et al. 2018). The FibroFatigue scale (FFS) was employed to evaluate fibromyalgia symptoms and chronic fatigue syndrome (Zachrisson et al. 2002). The Hamilton Anxiety Rating Scale (HAMA) was used to assess the severity of anxiety (Hamilton 1959). Body mass index (BMI) was calculated using the following formula: BMI = weight in kilograms / (height in meters)^2^.

### 2.3 Assays

Participants fasted for 8 h, and 25 mL of blood was collected from 6:30 to 7:30 a.m. The blood was divided into EDTA tubes (10 mL) and SST tubes (10 mL). SST tubes were left at room temperature for 30 min to clot, followed by centrifugation at 3500 rpm for 10 min at 4°C. Serum and plasma were separated and stored at −80°C.

In this study, we measured cytokines/chemokines/growth factors in the supernatant of whole blood cultures stimulated with PHA and LPS (Maes et al. 1999a). We used whole blood directly without prior PBMC isolation, as this preserves physiological cell–cell interactions and avoids activation artifacts that can occur during density‑gradient separation. The culture procedure was performed in 24‑well plates as follows: each well received 1.8 mL of RPMI‑1640 medium, 0.4 mL of PHA (5 µg/mL), 0.4 mL of LPS (25 µg/mL), and 20 µL of penicillin‑streptomycin solution (100 µg/mL). Then, 0.2 mL of EDTA‑anticoagulated whole blood was added to each well. Well 1, containing only the aforementioned reagents, served as the control group.

Ten milligrams of curcumin were diluted to obtain three concentrations: high (200 ng/mL, 542.92 nM), medium (20 ng/mL, 271.45 nM), and low (2 ng/mL, 5.43 nM) (Flory et al. 2021; Kroon et al. 2023). These concentrations were selected to span the range from physiological plasma levels to supra‑physiological exposures based on human pharmacokinetic data. The remaining wells were supplemented with high, medium, and low concentrations of curcumin solutions. The samples were incubated for 72 h in a humidified environment at 37°C and 5% CO_2_. After incubation, the samples were centrifuged at 1500 rpm for 8 min. Under sterile conditions, the supernatant was carefully removed, divided into Eppendorf tubes, and immediately frozen at −80°C until thawed for cytokine and growth factor detection.

The supernatants were used to detect 48 cytokines/chemokines/growth factors using the Bio‐Plex Pro Human Cytokine 48‐Plex Assay Kit (Bio‐Rad, Hercules, CA, USA). Cytokines/chemokines/growth factors were measured using the Luminex method, a multiplex detection method based on magnetic microbeads. In brief, supernatants were diluted 4‐fold with medium and incubated with conjugated magnetic microbeads for 30 min; then, detection antibodies were added and incubated for another 30 min, followed by 100× SA‐PE for 10 min. Finally, the fluorescence intensities (FI) were measured using the Luminex instrument. For statistical analysis, MFI‐blank values were employed, due to their greater reliability compared to absolute concentrations, especially when multiple plates were used (Breen et al. 2015). All cytokines/chemokine/growth factor assessments showed values higher than the limit of quantification values.

Table S2 lists all cytokines assessed in the present study, as well as their aliases and the number of samples with values lower than the sensitivity of the assay. Based on these measurements, we computed different immune profiles, including IRS, CIRS, M1 and M2, Th1, and Th2, growth factor, and chemokine, as well as profiles reflecting TNF and IL‐1 signaling (Almulla et al. 2024; Maes and Carvalho 2018). Based on these computed profiles, we estimated ratios between M1/M2 (reflecting M1 or M2 polarization), Th1/Th2 (reflecting Th1 or Th2 polarization), and the IRS/CIRS ratio, reflecting overall IRS activation or increased regulation (Almulla et al. 2024; Maes and Carvalho 2018). Table S3 shows how we computed these scores using *z*‐unit‐based composite scores.

### 2.4 Statistical Analysis

Analysis of variance (ANOVA) was used to assess differences in scale variables between groups, while chi‐square tests or Fisher's exact probability tests were used to compare categorical variables. A multivariate general linear model (GLM) was employed to compare serum concentrations of cytokines/chemokines/growth factors between patients with MDD and healthy controls, adjusting for age, sex, BMI, and plate number. Generalized estimating equation (GEE) analysis was used to examine repeated measurement data to evaluate the effects of curcumin treatment on cytokines/chemokines/growth factors. The GEE model included fixed effects of time (control vs. three treatments) and time × group (MDD vs. controls) interactions, with gender, age, BMI, and smoking status included as covariates. False discovery rate (FDR) was applied to adjust *p*‐values for multiple comparisons arising from main effects of time/group and their interactions on cytokines/chemokines/ growth factors profiles (Benjamini and Hochberg 1995). Multiple regression analysis was used to explore the associations between immune biomarkers and depression. The first principal component extracted from the HAMD, HAMA, and FFS was used as the dependent variable (severity of MDD), and the immune biomarkers served as explanatory variables, while controlling for the effects of sex, age, BMI, waistline, and education. All GLM and GEE models included age as a covariate to account for potential age‑related confounding. There were no missing values in any of the demographic, clinical, or cytokines/chemokines/growth factors data assessed in this study.

The primary outcome variables in our analysis were the immune profiles. Using GEE analyses, we examined the impact of curcumin on the profiles and the differences among MDD versus healthy controls. When there are significant effects, we consequently examine the separate cytokines/chemokines/growth factors. All statistical analyses were performed using IBM SPSS, version 29.0 for Windows.

The main analysis in this study consisted of repeated‐measurement analyses, which considered four repeated measurements (control vs. three curcumin concentrations) and two groups (healthy controls vs. MDD). Based on a priori minimal sample size to obtain an effect size of 0.25, alpha = 0.05, power = 0.8 is 24 for the within‐factor effects and 28 for the within‐between interactions.

## 3 Results

### 3.1 Differences in Immune Profiles Between MDD and Healthy Controls

**FIGURE 1:**
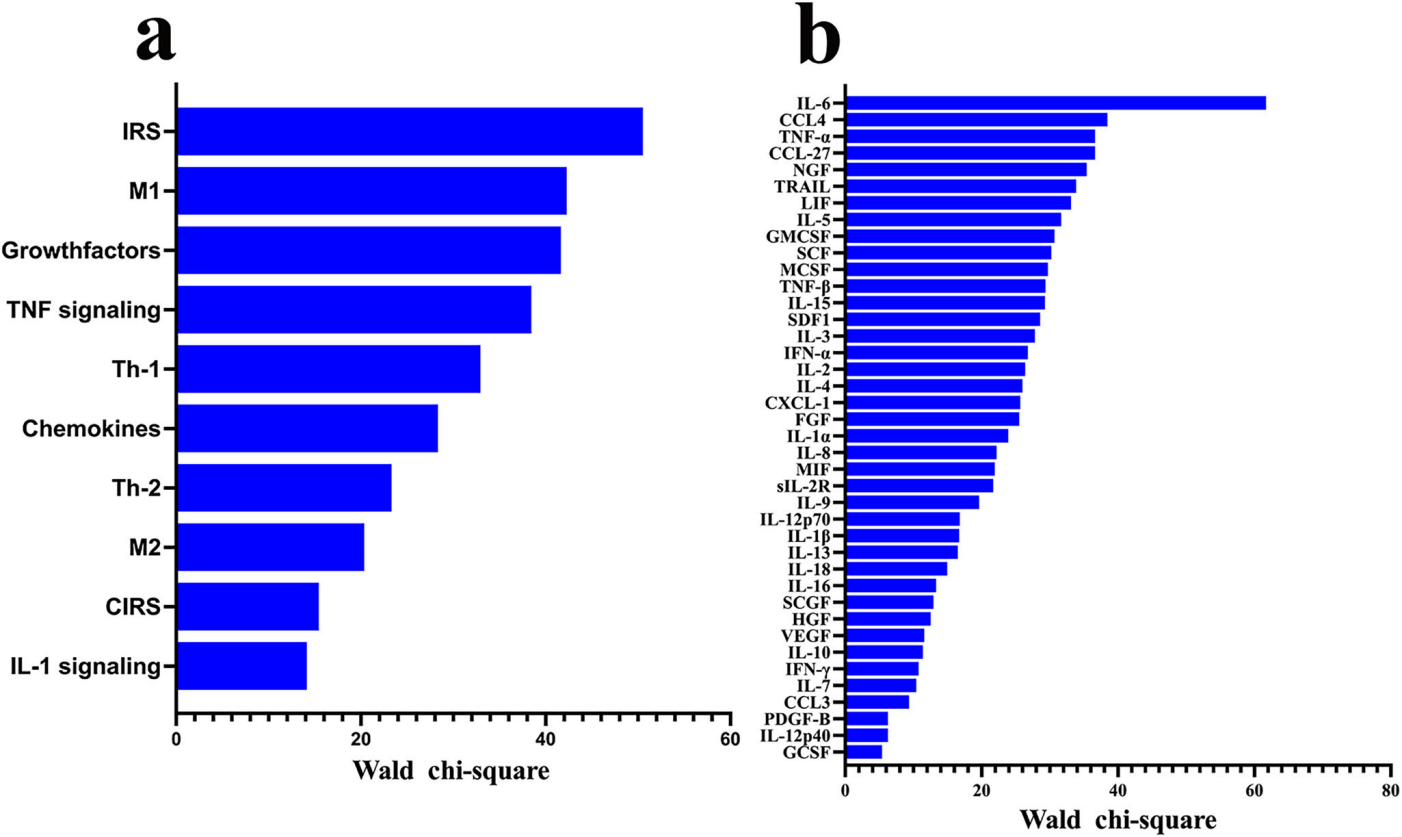
Differences in cytokines, chemokines, and growth factors between patients with major depressive disorder (MDD) and healthy controls. (a) Wald chi‐square values (displayed as a bar graph in descending order) comparing the composite immune profiles between MDD and HC. Statistical significance was assessed using a multivariate general linear model (GLM) with age, sex, BMI, and plate number as covariates. All displayed profiles remained significant after false discovery rate (FDR) correction (*p* < 0.05). (b) Wald chi‐square values for individual cytokines/chemokines showing significant group differences. The top seven biomarkers (IL‐6, CCL4, TNF‐α, CCL27, NGF, TRAIL, and LIF) exhibited the largest effect sizes.

Figure 1a demonstrates significant dysregulation across all measured immune profiles in MDD compared to HC, as quantified by Wald chi‐square statistics. While all cytokine/chemokine/growth factor categories showed statistically significant elevations in MDD, the most pronounced differences were observed in IRS, M1, growth factor, TNF signaling, and Th1 profiles (Table S4 for mean values). Differences were also evident in chemokines, Th2, M2, CIRS, and IL‐1 signaling profiles, albeit with a lower effect size. All those differences remained significant after FDR *p*‐correction (Table S4). Since the differences in immune profiles were significant, we consequently examined the solitary cytokines.

### 3.2 Differences in Solitary Cytokines Between MDD and Healthy Controls

As illustrated in Figure 1b, Wald chi‐square metrics are displayed as a bar graph revealing the significant differences in culture supernatant cytokines between MDD and controls. Tables S5 and S6 show that all cytokines were significantly higher in MDD than in controls, except for sIL‐1RA and CCL11. The top seven biomarkers (in descending order of importance were: IL‐6, CCL4, TNF‐α, CCL27, NGF, TRAIL, and LIF. Anti‐inflammatory cytokines, such as IL‐10 and sIL‐1A, showed less pronounced changes. Growth factors like VEGF displayed significant differences. All these differences remained significant after FDR *p*‐correction.

**FIGURE 2:**
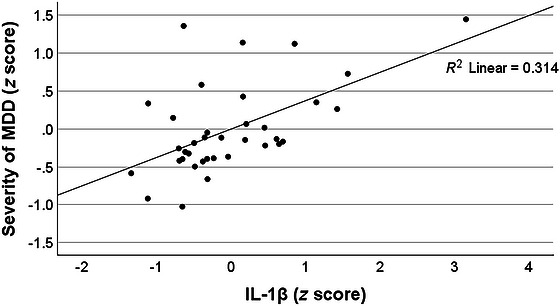
Partial regression of cytokines (IL‐1β) on the sum of Hamilton Depression Rating Scale (HAMD), Hamilton Anxiety Rating Scale (HAMA), and FibroFatigue scale (FFS). The dependent variable is the first principal component extracted from the total scores of the HAMD, HAMA, and FFS. IL‐1β (*z*‐scored) serves as the explanatory variable. The partial regression plot is based on all participants, with adjustments for age, sex, BMI, waist circumference, and education years.

We also analyzed the relationship between immune biomarkers and depression, using the principal component extracted from the total scores of HAMD, HAMA, and FFS as the dependent variable and immune biomarkers as the independent variable for partial regression analysis (Maes et al. 1994). For example, Figure 2 shows the results of a partial regression analysis using baseline data (before curcumin intervention) from all HC and MDD participants. IL‐1β exhibited significant positive correlations with depression severity.

Some patients were on a stable treatment regimen with antidepressants (*n* = 12, including mirtazapine, fluoxetine, sertraline, paroxetine, and others), mood stabilizers (*n* = 3), antipsychotics (*n* = 4), and benzodiazepines (*n* = 7). No significant effects of any of these drugs on the immune profiles or solitary cytokines could be found (even without FDR *p*‐correction).

### 3.3 Effects of Curcumin on Immune Profiles

We found two types of effects of curcumin on the immune data, namely treatment × group interactions and treatment effects without a significant interaction. These two types of responses are shown in different figures.

**FIGURE 3:**
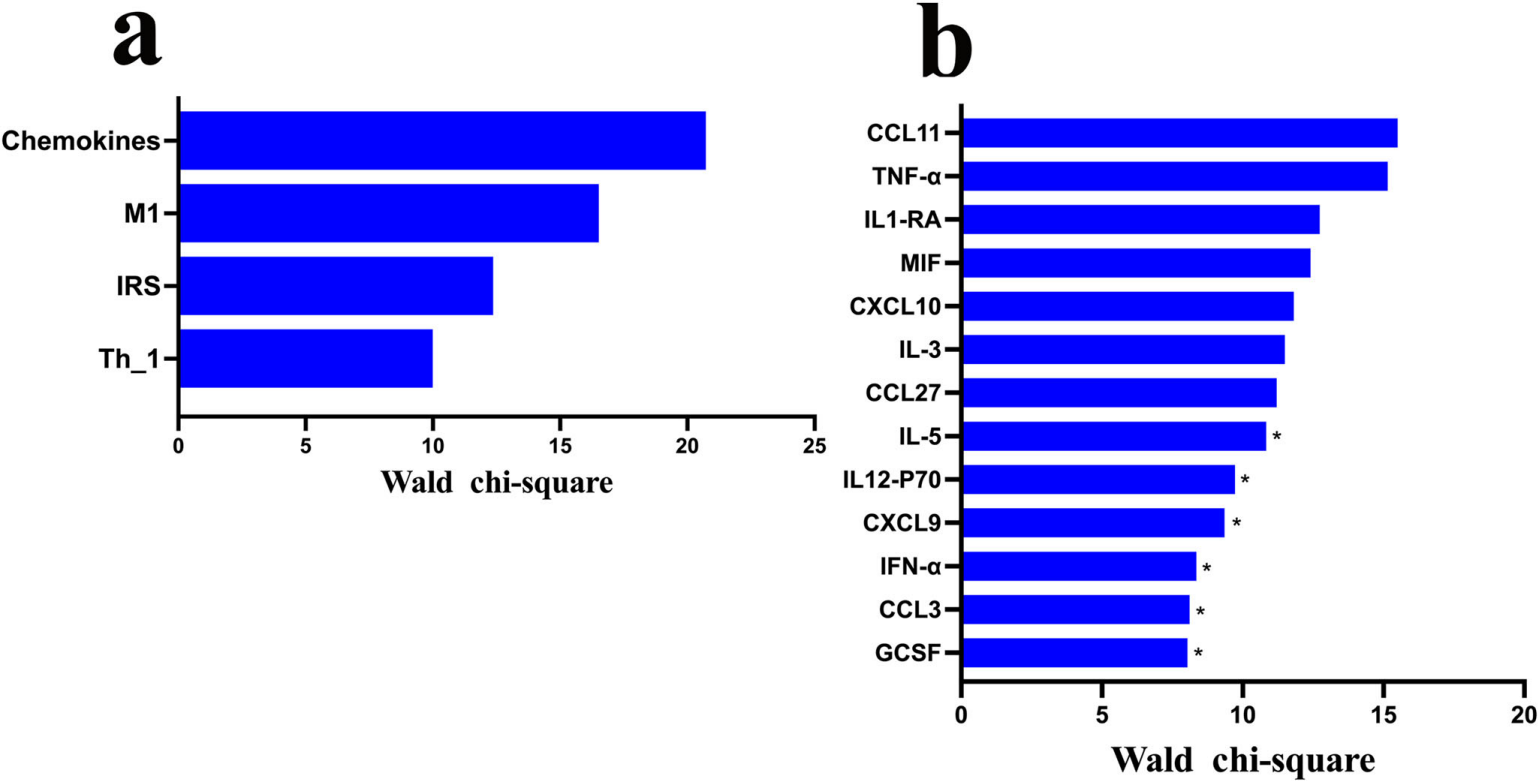
The significant group (major depression vs. controls) by treatment (control vs. three curcumin concentrations) interactions. (a) Wald chi‐square values for composite immune profiles showing significant interaction effects. (b) Wald chi‐square values for individual cytokines/chemokines with significant group × treatment interactions. All interactions were assessed using generalized estimating equations (GEE) with group, treatment, and group × treatment as fixed effects, and age, sex, BMI, and smoking as covariates. *Non‐significant after false‐discovery rate *p*‐correction.

**FIGURE 4:**
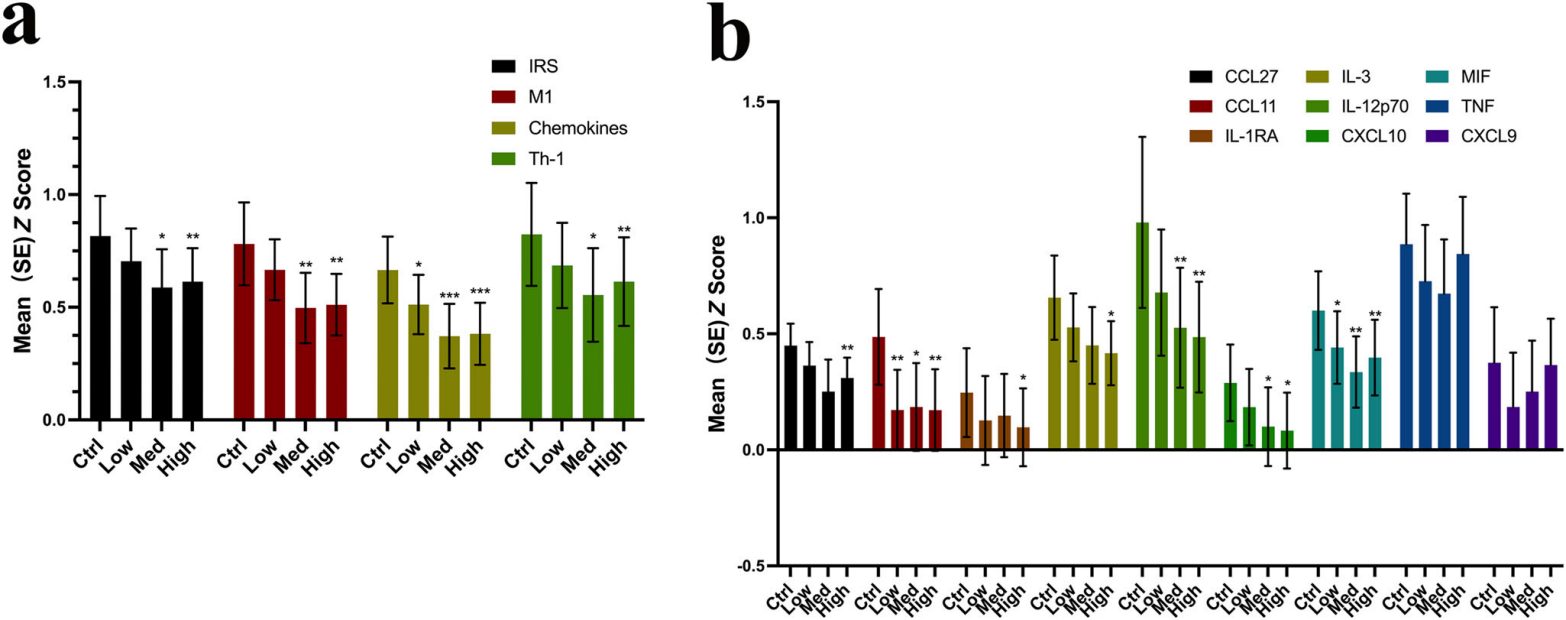
Effects of different concentrations of curcumin on cytokines, chemokines, and growth factors in patients with major depression. (a) Treatment effects (pooled across the three curcumin concentrations) showing significant suppression of the overactivated IRS, M1, Th1, and chemokine profiles in MDD patients (GEE main effect of treatment; *n* = 72 per group). (b) Box plots showing the dose‐dependent suppressive effects of curcumin on nine individual mediators (CCL27, CCL11, IL‐1RA, IL‐3, IL‐12p70, CXCL10, MIF, TNF‐α, and CXCL9) in MDD patients. *p < 0.05, **p < 0.01, ***p < 0.001, ****p < 0.0001. Ctrl: control, Low: low‐dose curcumin (2 ng/mL), Med: med‐dose curcumin (20 ng/mL), High: high‐dose curcumin (200 ng/mL).

Figure 3a shows that the interactions between treatment × group were significant for four immune profiles, namely chemokines, M1, IRS, and Th1. Figure 4a demonstrates that curcumin exerts significant effects on these immune profiles. The differences remained significant after FDR *p*‐correction. We could not find any significant effects of curcumin treatment (either fixed effects of treatment or interactions group × treatment) on CIRS, M2, Th2, IRS/CIRS ratio, M1/M2 ratio, and TNF and IL‐1 signaling profiles.

### 3.4 Effects of Curcumin on Separate Cytokines

As shown in Figure 3b, cytokines/chemokines such as CCL11, TNF‐α, IL‐1RA, MIF, CXCL10, IL‐3, CCL27, and IL‐5 exhibited the most pronounced interactions (highest Wald *χ* ^2^ values). Therefore, we have analyzed these cytokines/chemokines in MDD and controls separately. After FDR *p*‐correction, CCL11, TNF‐α, IL‐1RA, MIF, CXCL10, IL‐3, and CCL27 remained significant, whereas IL‐5, IL‐12p70, CXCL9, IFN‐α, CCL3, and G‐CSF were no longer significant. In Figure 4b, we show the suppressant effects of curcumin administration in MDD patients on nine of these cytokines, namely CCL27, CCL11, IL‐1RA, IL‐3, IL‐12p70, CXCL10, MIF, TNF‐α, and CXCL9.

**FIGURE 5:**
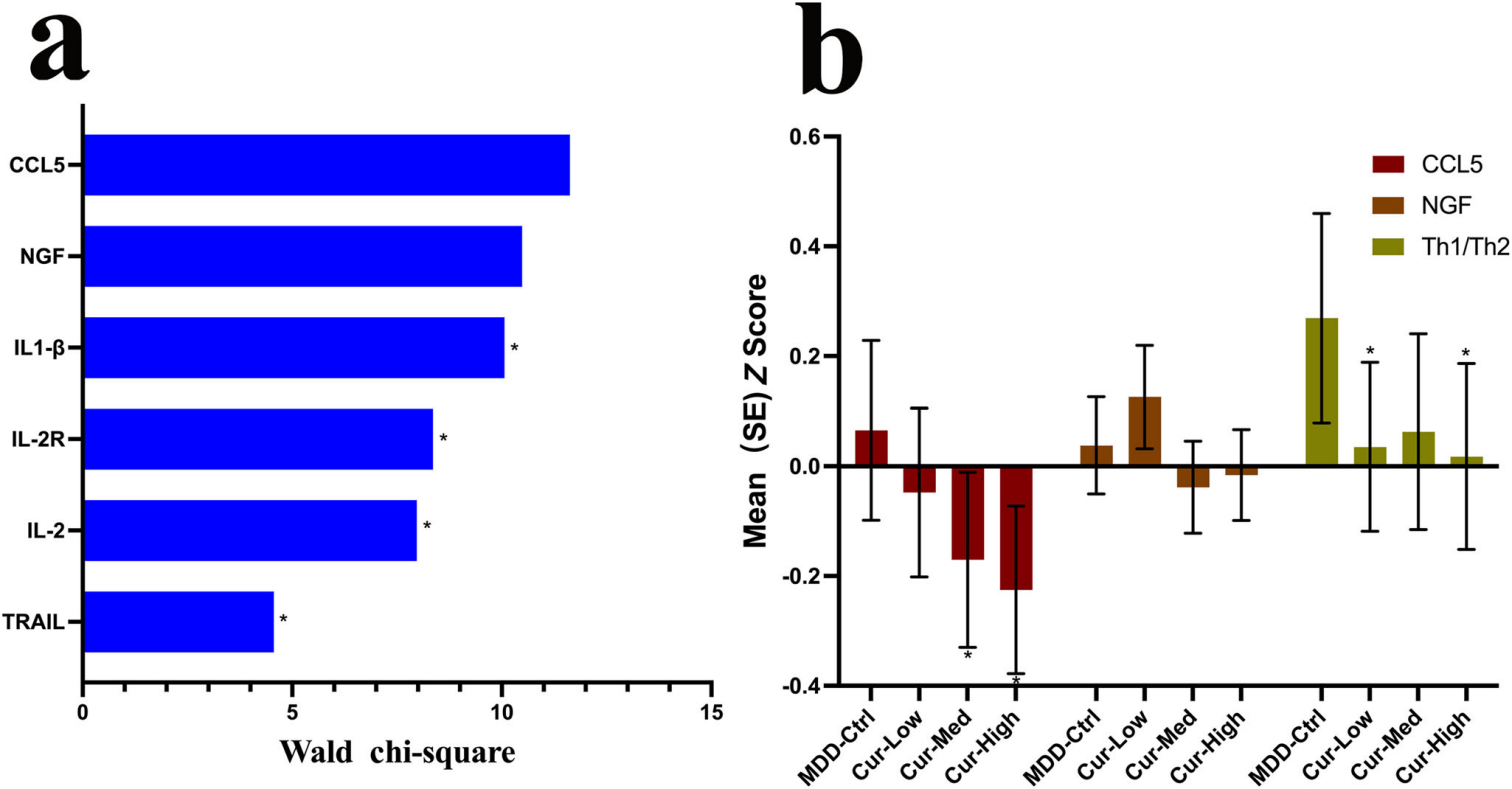
The treatment effects of curcumin (control vs. three curcumin concentrations) without a significant group by treatment interaction. (a) Wald chi‐square values for the main effect of treatment (curcumin vs. control) on CCL5, NGF, IL‐1β, IL‐2R, IL‐2, and TRAIL. *Non‐significant after false‐discovery rate *p*‐correction. (b) Effects of different concentrations of curcumin on cytokines, chemokines, and growth factors in patients with major depression.*p < 0.05, **p < 0.01, ***p < 0.001, ****p < 0.0001. Ctrl: control, Low: low‐dose curcumin (2 ng/mL), Med: med‐dose curcumin (20 ng/mL), High: high‐dose curcumin (200 ng/mL).

Figure 5 shows that curcumin had significant effects without a significant treatment × group interaction on CCL5, NGF, IL‐1β, IL‐2R, IL‐2, and TRAIL. After FDR *p*‐correction, IL‐1β, IL‐2R, IL‐2, and TRAIL were no longer significant. In addition, curcumin treatment significantly decreased the Th1/Th2 ratio (*p* = 0.016) without a significant interaction effect. Figure 5b shows the treatment effects of curcumin administration on CCL5 and Th1/Th2 ratio (suppressant) and NGF (enhanced).

There were no significant effects of curcumin (either via treatment or treatment × group interactions) on the following cytokines: IL‐1α, IL‐4, IL‐6, IL‐7, IL‐8, IL‐10, IL‐12p40, IL‐13, IL‐15, IL‐16, IL‐17, IL‐18, IFN‐γ, LIF, and TNF‐β. Curcumin did not affect chemokines such as CCL2, CCL4, CCL7, CXCL1, and CXCL12; colony‐stimulating factors such as GM‐CSF and M‐CSF, and growth factors such as FGF, HGF, PDGF, CSGF, SCF, and VEGF.

**FIGURE 6:**
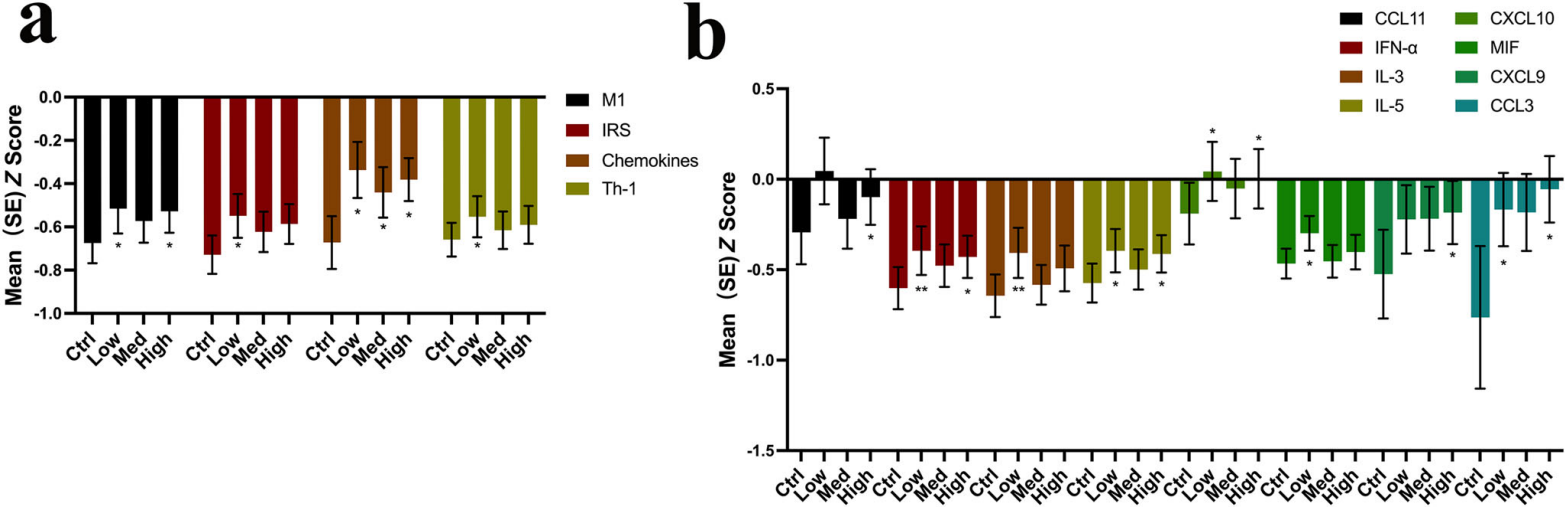
Effects of different doses of curcumin on cytokines, chemokines, and growth factors in healthy controls. (a)Treatment effects (pooled across the three concentrations) showing a modest but significant upward trend in M1, IRS, chemokine, and Th1 profiles following curcumin administration in HC. (b) Box plots showing the consistent upregulation of individual chemokines/cytokines (CCL11, IFN‐α, CXCL10, MIF, CXCL9, and CCL3) after curcumin treatment in HC. *p < 0.05, **p < 0.01, ***p < 0.001, ****p < 0.0001. Ctrl: control, Low: low‐dose curcumin (2 ng/mL), Med: med‐dose curcumin (20 ng/mL), High: high‐dose curcumin (200 ng/mL).

In contrast, curcumin showed quite a different pattern in the HC group (Figure 6). Following curcumin intervention, the four immune profiles (M1, IRS, chemokines, and Th1) exhibited a moderate upward trend (Figure 6a). Similarly, CCL11, IFN‐α, CXCL10, MIF, CXCL9, and CCL3 showed a slight but consistent upregulation after curcumin administration (Figure 6b).

## 4 Discussion

### 4.1 Differences in Immune Profiles in MDD Versus HC

MDD is marked by immune sensitization, where both IRS and CIRS profiles show heightened reactivity. Our ex vivo whole‐blood assays reveal significant elevations across all measured immune profiles in MDD patients versus HC, with the most pronounced differences in IRS, M1 macrophages, Th1 responses, and TNF signaling pathways. This aligns with the immune sensitization hypothesis proposed by Maes et al. (2022), who suggest that immune cells in MDD are in a primed, hyperresponsive state. Clinical in vivo evidence supports this, with meta‐analyses confirming elevated serum/plasma levels of pro‐inflammatory cytokines (e.g., IL‐6, TNF‐α, and IFN‐γ) in MDD (Dowlati et al. 2010; Köhler et al. 2017), and increased M1 macrophage/Th1 activity and impaired CIRS function (e.g., reduced Treg/Th2 responses) further indicating immune imbalance (Almulla et al. 2024; Köhler et al. 2018). All primary conclusions in this study are drawn exclusively from findings that remained significant after FDR correction. Trends that were significant only at the uncorrected level are not interpreted as meaningful effects and are not included in our main findings.

Importantly, immune sensitization and serum cytokine levels are distinct phenomena. Serum cytokine elevations reflect systemic inflammatory outputs, serving as downstream markers of immune activation. In contrast, immune sensitization denotes a hyperreactive cellular state, evidenced by exaggerated cytokine production upon *ex vivo* immunogen challenge (e.g., LPS/PHA) (Figure 1). The latter sensitization is a hallmark of MDD (Debnath et al. 2021). Our data extend this paradigm by demonstrating that sensitization encompasses both IRS and CIRS pathways. While IRS activation (e.g., IL‐6, TNF‐α) dominated, CIRS components (e.g., IL‐10, Th2) also showed elevated reactivity—albeit insufficient to counterbalance IRS‐driven inflammation. This aligns with the IRS‐CIRS imbalance theory, where CIRS failure permits net IRS hyperactivation in MDD (Maes et al. 2022).

In summary, MDD exhibits broad immune sensitization across various immune profiles, with IRS hyperactivity as a dominant feature. This immune sensitization—representing the inducible cytokine‐producing capacity of immune cells, as distinct from basal circulating cytokine concentrations—may constitute an important mechanism contributing to neuroinflammation and the clinical severity of MDD (Maes et al. 2025).

### 4.2 Curcumin Selectively Suppresses Overactivated Inflammatory Pathways in MDD

Our ex vivo findings demonstrate that curcumin exerts clear and pathway‐selective immunomodulatory effects on stimulated immune cells from MDD patients. Curcumin significantly downregulated key IRS‐associated immune profiles, including chemokine, IRS, M1, and Th1 profiles, indicating its capacity to precisely target the core overactivated inflammatory pathways in MDD.

Moreover, in MDD patients, curcumin significantly suppressed multiple pro‐inflammatory mediators, including cytokines (TNF‐α, IL‐3, and IL‐1RA), chemokines (CCL5, CCL27, CXCL10, and CCL11), and macrophage migration inhibitory factor (MIF), while stimulating the production of neuroprotective nerve growth factor (NGF). These results suggest that curcumin exhibits its strongest effects on the chemokine network in MDD patients, which may have positive implications for mitigating neuroinflammation. Notably, curcumin did not enhance CIRS profiles (including M2 and Th2), indicating that its mechanism is primarily centered on suppressing overactivated inflammatory pathways rather than broadly restoring immune homeostasis—a factor that may explain its limited efficacy as monotherapy.

This efficacy of curcumin at reducing immune sensitization aligns with evidence from non‐depressive human models showing that curcumin inhibits LPS‐induced production of IL‐1β, IL‐6, and TNF‐α in monocytes/macrophages (Ghany et al. 2023), and animal depression models where it suppresses neuroinflammation by inhibiting NF‐κB, reducing hippocampal pro‐inflammatory cytokines, and blocking NLRP3 inflammasome activation (Fan et al. 2021).

### 4.3 Clinical Implications of Curcumin as an Adjunctive Therapy in MDD

Curcumin demonstrates efficacy as an augmentation strategy, potentially reversing MDD progression (Yu et al. 2015), and is safe and well‐tolerated alongside antidepressants. Its therapeutic impact is significantly enhanced in combination with agents like piperine (Panahi et al. 2015) or saffron (Lopresti and Drummond 2017), and meta‐analyses confirm that adjunctive curcumin improves depression and anxiety symptoms in MDD (Kanchanatawan et al. 2018). The absence of a statistical interaction between the effects of in vivo antidepressant treatment and in vitro curcumin administration does not exclude the possibility that both interventions exert complementary or synergistic immunomodulatory effects in vivo. We hypothesize that curcumin may act through additional mechanisms not captured in our whole‑blood culture: (i) pharmacokinetic enhancement of antidepressant bioavailability; (ii) gut‑microbiome modulation reducing endotoxin‑induced immune activation; and (iii) neuroplasticity and antioxidant effects via BDNF and Nrf2 pathways (Liao et al. 2020; Zhang et al. 2019). These pathways warrant future in vivo investigation. Our findings further provide a mechanistic basis for its clinical efficacy: the antidepressant effects of curcumin may be at least partially attributed to its selective inhibition of core inflammatory pathways (particularly IRS/M1/Th1/chemokines) in MDD. Although curcumin does not fully reverse the immune imbalance in MDD, its significant downregulation of key inflammatory mediators is likely sufficient to produce meaningful clinical benefits.

The partial efficacy observed ex vivo and the requirement for prolonged administration in vivo may stem from curcumin's multi‐target actions. These include transcriptional regulation of enzymes (downregulating COX‐2 and 5‐LOX), modulation of gut‐immune crosstalk (restoring intestinal barrier integrity and microbiome balance), and potent antioxidant activity (scavenging ROS that potentiate NLRP3 activation) (Chen et al. 2019; Dilmore et al. 2023; Hong et al. 2004; Patel et al. 2020). Moreover, curcumin directly promotes neuroplasticity. In rodent models of depression (CUMS, LPS‐induced), it increases the pCREB/CREB ratio, elevates BDNF and PSD‐95 expression, mitigates oxidative stress and neuronal apoptosis, prevents synaptic loss in the hippocampus, and alleviates reductions in dendritic spine density and length (Liao et al. 2020; Zhang et al. 2019). These neuroprotective and plasticity‐enhancing effects contribute to its overall antidepressant action.

The modest but significant immunomodulation, particularly against M1/Th1 hyperactivation, may contribute to the clinical efficacy of curcumin. Clinical trials consistently report that curcumin supplementation (typically 500–1000 mg/day) reduces serum pro‐inflammatory cytokines (IL‐6, TNF‐α) and improves depressive symptoms, but these benefits become evident only after 4–8 weeks, with substantial improvement requiring 12–16 weeks (Lopresti et al. 2014). The ex vivo data suggest that this delayed clinical efficacy reflects the time needed for the cumulative, pathway‐specific effects to translate into measurable symptom reduction.

While curcumin exhibits significant anti‐inflammatory and neuroprotective properties relevant to MDD pathophysiology, its incomplete restoration of IRS‐CIRS equilibrium and failure to fully reverse immune sensitization highlight a key limitation. Its immunoregulatory effects, focused primarily on suppressing IRS/M1/Th1 hyperactivity with minimal enhancement of compensatory CIRS responses, appear insufficient as monotherapy for fully resolving the complex immune dysfunction in MDD. This underscores the biological rationale for the observed superior efficacy of combinatorial strategies, such as co‐administration with piperine, saffron, or standard antidepressants. Future therapeutic development should focus on augmenting curcumin's IRS‐suppressive effects with agents that actively promote CIRS‐mediated immunoregulation.

### 4.4 Effect of Curcumin on Immune Profiles in Controls

Our ex vivo findings reveal a paradoxical effect of curcumin in healthy individuals: curcumin significantly increased key immune profiles, including M1 macrophages, IRS pathways, chemokines, Th1 responses, and some chemokines (e.g., CCL11, CXCL10, CCL3, and CXCL9). This contrasts with its immunoregulatory effects in MDD patients.

To our knowledge, this immune‐stimulating effect of curcumin in immunologically quiescent individuals is a novel finding. While curcumin is widely regarded as safe in healthy populations, its ex vivo induction of IRS and chemokine responses—particularly neurotoxic and pro‐inflammatory mediators like CCL11 (linked to neuroinflammation and cognitive impairment) (Fernández‐Castañeda et al. 2022), CXCL10 (associated with BBB disruption and neuroinflammation) (Jorfi et al. 2023), and CCL3 (a potent neuroinflammatory chemokine) (Wang et al. 2024)—suggests potential adverse implications for neuronal health and immune homeostasis. Our findings suggest that curcumin may not universally function as a beneficial “anti‐inflammatory supplement” for individuals without pre‐existing immune issues. Our ex vivo observations suggest a potential immune‑stimulating effect in healthy individuals, which warrants caution and further clinical investigation before any recommendations regarding prophylactic use can be made.

## 5 Limitations

This study has several limitations. First, the ex vivo whole‐blood assay model, while useful for assessing immune sensitization, may not fully replicate the in vivo complexity of the immune‐brain axis. Second, although the study was well powered, the sample size may limit the statistical power for subgroup analyses (e.g., TRD, increased BMI). Third, some participants were concurrently taking psychotropic medications (e.g., SSRIs, antipsychotics, benzodiazepines). No significant differences were observed between drug‐free and medicated patients for any immune profile or individual cytokine. As medication status constitutes the primary and more comprehensive source of treatment‐related variability, these findings argue against confounding by medication dose or treatment duration. Future longitudinal studies in drug‑free MDD patients would be valuable to confirm the immunomodulatory effects of curcumin observed here and to examine its long‑term effects on cytokine networks.

Fourth, although age was closely matched between groups and included as a covariate in all statistical models, the modest sample size precluded formal age‐stratified analyses or the evaluation of non‐linear age‐related effects, including potential influences of the senescence‐associated secretory phenotype (SASP). Fifth, BMI, rather than BIA, was used as a surrogate measure of adiposity. Although BIA provides a more accurate assessment of body fat, adjustment for BMI did not affect the reported associations. It would be interesting if future research could measure intracellular signaling molecules such as NF‑κB or Nrf2. Future studies using isolated PBMCs could elucidate these upstream pathways, as our whole‑blood system is less suited for reliable intracellular assays.

Although we applied FDR correction to control for false positives due to multiple comparisons, we acknowledge that the relatively modest sample size may limit the power to detect small‑to‑moderate effects. Consequently, we have conservatively restricted our interpretation to FDR‑confirmed findings and have not concluded uncorrected trends.

## 6 Conclusions

This study demonstrates that curcumin selectively suppresses overactivated core inflammatory pathways in MDD patients, including IRS, M1, Th1, and multiple pro‐inflammatory chemokines (e.g., CCL11 and CXCL10), thereby partially alleviating immune sensitization. Although curcumin did not enhance CIRS profiles, its significant downregulation of key pro‐inflammatory mediators provides an important mechanistic basis for its clinical antidepressant efficacy. Notably, curcumin exhibited mild immune‐stimulating effects in healthy controls, warranting caution regarding its prophylactic use in immunologically quiescent individuals.

These findings support curcumin as a promising adjunctive immunomodulator for MDD, with its selective anti‐inflammatory actions likely contributing to its clinical benefits. However, its limited efficacy as monotherapy suggests that future research should explore combination strategies targeting both IRS suppression and CIRS enhancement to achieve more comprehensive immune homeostasis. Clinically, curcumin is a reasonable adjunctive option for MDD based on our mechanistic data and prior clinical evidence. However, its ex vivo immune‑stimulating effect in healthy controls warrants caution, and further in vivo studies are needed before any recommendations regarding prophylactic use in immunologically healthy individuals can be made.

## Author Contributions

All the contributing authors have participated in the manuscript. M.M. and Y.Z. designed the study. Y.Z., T.C., Y.L., and M.L performed the assays. T.C., Y.L., M.M., and Y.Z. wrote the manuscript. M.N. and J.L. recruited the patients. All authors have read and agreed to the published version of the manuscript.

## Funding

This research was funded by the Sichuan Science and Technology Program “PIANJI” Project (Grant No. 2025HJPJ0004).

## Ethics Statement

This study was approved by the Ethics Committee of the University of Electronic Science and Technology of China (Approval No. 30005) and strictly followed ethical and privacy regulations. It adhered to the standards outlined in the Belmont Report and the International Conference on Harmonization in Good Clinical Practice (ICH‐GCP).

## Conflicts of Interest

The authors declare no conflicts of interest.

## Supporting information




**Supplementary Information**: brb371672‐sup‐0001‐TableS1‐S6.docx

## Data Availability

Data will be provided by the senior author (M.M.) upon a reasonable request.
