# Supplementary material for: Selective Anti‐Inflammatory Actions of Curcumin on Cytokine Pathways in Major Depressive Disorder
Source: Brain Behav. 2026 Aug 12;16(8):e71672. doi: 10.1002/brb3.71672 (PMC13463131; doi:10.1002/brb3.71672)
Supplement: Supplementary file 1 [file BRB3-16-e71672-s001.docx]

**Supplementary File**

***Selective Anti-Inflammatory Actions of Curcumin on Cytokine Pathways in Major Depressive Disorder***

Tangcong Chen^1, 2#^, Yueyang Luo^1, 2#^, Mengqi Niu^1, 2^, Jing Li ^1, 2^, Mengdie Li^1, 2^, Yingqian Zhang^1, 2*^, Michael Maes^1-6*^

#Tangcong Chen and Yueyang Luo contributed equally to this work.

1 Sichuan Provincial Center for Mental Health, Sichuan Provincial People’s

Hospital, School of Medicine, University of Electronic Science and Technology of China, Chengdu 610072, China.

2 Key Laboratory of Psychosomatic Medicine, Chinese Academy of Medical Sciences, Chengdu 610072, China.

3 Department of Psychiatry, Medical University of Plovdiv, Plovdiv, Bulgaria.

4 Research Institute, Medical University Plovdiv, Plovdiv, Bulgaria.

5 Research and Innovation Program for the Development of MU - PLOVDIV (SRIPD-MUP), Creation of a network of research higher schools, National Plan for Recovery and Sustainability, European Union – NextGenerationEU, Medical University of Plovdiv, Plovdiv, Bulgaria.

6 Kyung Hee University, 26 Kyungheedae-ro, Dongdaemun-gu, Seoul 02447, Republic of Korea.

*Co-Corresponding authors:

Prof. Dr. Michael Maes, M.D., Ph.D. and Yingqian Zhang

Sichuan Provincial Center for Mental Health

Sichuan Provincial People’s Hospital,

School of Medicine,

University of Electronic Science and Technology of China

Chengdu 610072

China

Email: michaelmaes@uestc.edu.cn

ORCID ID: https://orcid.org/0000-0002-2012-871X

Michael Maes Google Scholar profile

**Supplementary Table 1: Demographic and clinical data of MDD patients and healthy controls.**

| **Variables** | **HC**  **(n=18)** | **MDD**  **(n=18)** | **F/**$\boldsymbol{\chi}$**²** | **df** | ***P*** |
| --- | --- | --- | --- | --- | --- |
| Age (Years) | 38.61 (14.1) | 38.22 (11.22) | 0.008 | 1/34 | 0.928 |
| Gender (Male/Female) | 6/12 | 6/12 | 0.000 | 1 | 1 |
| BMI (kg/m²) | 23.86 (2.90) | 21.27 (3.20) | 6.458 | 1/34 | 0.016 |
| Education year | 13.78 (3.782) | 13.94 (4.065) | 0.476 | 1/34 | 0.495 |
| Married (yes/no) | 9/9 | 13/5 | 1.870 | 1 | 0.171 |
| Smoking (yes/no) | 0/18 | 6/12 | 7.200 | 1 | 0.019 |
| Drink(yes/no) | 4/14 | 4/14 | 0.000 | 1 | 1 |
| HAMD | 1.61(1.883) | 27.33 (5.041) | MWUT | - | < 0.001 |
| HAMA | 0.93 (1.072) | 22.35 (6.864) | MWUT | - | < 0.001 |
| FFS | 1.88 (2.804) | 32.28 (10.637) | MWUT | - | < 0.001 |

Data are shown as mean (SD) for continuous variables and as counts for categorical variables. Group comparisons were performed using analysis of variance (ANOVA) for continuous variables (age, BMI, education) and chi‑square (χ²) or Fisher’s exact test for categorical variables (gender, marital status, smoking, drinking). For HAMD, HAMA, and FFS scores, the MWUT was used due to non‑normal distribution.

HC: Healthy control; MDD: major depressive disorder; BMI: body mass index; MWUT: Mann-Whitney U test; HAMD: Hamilton depression rating scale; HAMA: Hamilton anxiety rating scale; FFS: Fibro Fatigue Scale;

**Supplementary Table 2**. **Overview of the cytokines, chemokines, and growth factors measured in the current study.**

| **Protein Abbreviations** | **Protein Name** | **Gene Symbol** | **Number of samples that lower than sensitivity of the assay** |
| --- | --- | --- | --- |
| IFN-α2 | Interferon alpha 2 | IFNA2 | 0 |
| IFN-γ | Interferon gamma | IFNG | 0 |
| IL-1α | Interleukin-1 alpha | IL1A | 0 |
| IL-1β | Interleukin-1 beta | IL1B | 0 |
| IL-1ra | Interleukin-1 receptor antagonist | IL1RN | 0 |
| IL-2 | Interleukin-2 | IL2 | 0 |
| IL-2Rα | Interleukin-2 receptor alpha | IL2RA | 0 |
| IL-3 | Interleukin-3 | IL3 | 0 |
| IL-4 | Interleukin-4 | IL4 | 0 |
| IL-5 | Interleukin-5 | IL5 | 0 |
| IL-6 | Interleukin-6 | IL6 | 0 |
| IL-7 | Interleukin-7 | IL7 | 0 |
| IL-8 (CXCL8) | Interleukin-8 | CXCL8 | 0 |
| IL-9 | Interleukin-9 | IL9 | 0 |
| IL-10 | Interleukin-10 | IL10 | 0 |
| IL-12 (p70) | Interleukin-12 | IL12A | 0 |
| IL-12 (p40) | Interleukin-12 (p40) | IL12B | 0 |
| IL-13 | Interleukin-13 | IL13 | 0 |
| IL-15 | Interleukin-15 | IL15 | 0 |
| IL-16 | Interleukin-16 | IL16 | 0 |
| IL-17A | Interleukin-17A | IL17A | 0 |
| IL-18 | Interleukin-18 | IL18 | 0 |
| TNF-α | Tumor necrosis factor | TNF | 0 |
| TNF-β | Tumor necrosis factor beta | LTA | 0 |
| TRAIL | TNF-related apoptosis-inducing ligand | TNFSF10 | 0 |
| LIF | Leukemia inhibitory factor | LIF | 0 |
| MIF | Macrophage migration inhibitory factor | MIF | 0 |
| G-CSF | Colony stimulating factor 3 | CSF3 | 0 |
| M-CSF | Macrophage colony stimulating factor | CSF1 | 0 |
| GM-CSF | Colony stimulating factor 2 | CSF2 | 0 |
| CCL2 (MCP-1) | C-C motif chemokine ligand 2 | CCL2 | 0 |
| CCL3 (MIP-1α) | C-C motif chemokine ligand 3 | CCL3 | 0 |
| CCL4 (MIP-1β) | C-C motif chemokine ligand 4 | CCL4 | 0 |
| CCL5 (RANTES) | C-C motif chemokine ligand 5 | CCL5 | 0 |
| CCL7 (MCP-3) | C-C motif chemokine ligand 7 | CCL7 | 0 |
| CCL11 (Eotaxin) | C-C motif chemokine ligand 11 | CCL11 | 0 |
| CCL27 (CTACK) | C-C motif chemokine ligand 27 | CCL27 | 0 |
| CXCL1 (GRO-α) | C-X-C motif chemokine ligand 1 | CXCL1 | 0 |
| CXCL9 (MIG) | C-X-C motif chemokine ligand 9 | CXCL9 | 0 |
| CXCL10 (IP-10) | C-X-C motif chemokine ligand 10 | CXCL10 | 0 |
| CXCL12 (SDF-1α) | C-X-C motif chemokine ligand 12 | CXCL12 | 0 |
| Basic FGF | Basic fibroblast growth factor 2 | FGF2 | 0 |
| HGF | Hepatocyte growth factor | HGF | 0 |
| β-NGF | Beta nerve growth factor | NGF | 0 |
| PDGF-B | Platelet derived growth factor subunit B | PDGFB | 0 |
| SCF | Stem cell factor | KITLG | 0 |
| VEGF | Vascular endothelial growth factor A | VEGFA | 0 |
| SCGF-β | Stem cell growth factor beta | CLEC11A | 0 |

**Supplementary Table 3**. **Description of the immune profiles used in this study.**

| Immune Profile | Members |
| --- | --- |
| M1 macrophage | IL-1β, IL-6, TNF-α, IL-12p70, IL-15, CCL2, CCL5, CXCL1, CXCL8, CXCL9, CXCL10 |
| M2 macrophages | IL-10, IL-4, IL-13, VEGF, PDGF, sIL-1RA |
| M1 / M2 | zM1 – zM2 |
| T helper (Th)-1 | IL-2, sIL-2R, IFN-a, IFN-γ, IL-12p70, IL-16, TNF-α, TNF-β |
| Th-2 | IL-4, IL-5, IL-9, IL-13, IL-10, IL-6 |
| Th-1- z Th-2 | zTh-1 – zTh-2 |
| IRS | IL-1α, IL-1β, IL-6, TNF-α, IL-12p70, IL-15, IL-16, IL-17, IL-18, CCL2, CCL3, CCL4, CCL5, CCL7, CCL11, CXCL1, CXCL8, CXCL9, CXCL10, IL-2, IFN-α, IFN-γ, TNF-α, TNF-β, TRAIL, GM-CSF, M-CSF, G-CSF, SCGF |
| CIRS | IL-4, IL-10, sIL-1RA, sIL-2R |
| IRS/CIRS | Z IRS – z CIRS |
| Chemokines | CCL2, CCL3, CCL4, CCL5, CCL7, CCL11, CCL27, CXCL1, CXCL8, CXCL9, CXCL10, CXCL12 |
| Growth factors | Basic FGF, HGF, β-NGF, PDGF-BB, SCF, VEGF, SCGF-β, M-CSF, G-CSF, GM-CSF |
| TNF | TNF-α, TNF-β, TRAIL, LIF |
| IL-signaling | IL-1α, IL-1β, IL-1ra, IL-2, IL-2Rα, IL-3, IL-4, IL-5, IL-6, IL-7, IL-9, IL-10, IL-12 (p70), IL-12 (p40), IL-13, IL-15, IL-16, IL-17A, IL-18 |

**IRS**: immune-inflammatory response system; **CIRS**: compensatory immunoregulatory system

**Supplementary Table 4**. **Differences in immune cells and immune profiles between major depressive disorder (MDD) and healthy controls (HC).**

| Variables (z scores) | HC (n=72) | MDD (n=72) | χ² | df | p-value |
| --- | --- | --- | --- | --- | --- |
| z IRS – z CIRS | –0.344 (0.111) | 0.527 (0.203) | 14.096 | 1 | < 0.001 |
| IRS | –0.622 (0.086) | 0.681 (0.153) | 50.527 | 1 | < 0.001 |
| CIRS | –0.471 (0.111) | 0.463 (0.197) | 15.434 | 1 | < 0.001 |
| M1 | –0.573 (0.095) | 0.614 (0.145) | 42.279 | 1 | < 0.001 |
| M2 | –0.509 (0.090) | 0.551 (0.212) | 20.346 | 1 | < 0.001 |
| M1 – M2 | 0.470 (0.042) | 0.556 (0.051) | 1.842 | 1 | 0.190 |
| Th-1 | –0.604 (0.082) | 0.669 (0.200) | 32.953 | 1 | < 0.001 |
| Th-2 | –0.546 (0.106) | 0.575 (0.193) | 23.315 | 1 | < 0.001 |
| Th-1 – Th-2 | –0.208 (0.121) | 0.400 (0.275) | 5.058 | 1 | 0.028 |
| chemokines | –0.458 (0.100) | 0.483 (0.135) | 28.341 | 1 | < 0.001 |
| growth factors | –0.579 (0.085) | 0.651 (0.167) | 41.646 | 1 | < 0.001 |
| TNF-signaling | –0.653 (0.079) | 0.775 (0.224) | 38.457 | 1 | < 0.001 |
| IL-1 signaling | –0.520 (0.133) | 0.515 (0.214) | 14.137 | 1 | < 0.001 |

**M:** macrophage; **Th:** T helper; **IRS:** immune-inflammatory response system; **CIRS:** compensatory immunoregulatory response system.

**Supplementary Table 5**. **Differences in cytokines between major depressive disorder (MDD) and healthy controls (HC).**

| **Variables (z scores)** | **HC (n=72)** | **MDD (n=72)** | **Wald Chi-square** | **df** | **p-value** |
| --- | --- | --- | --- | --- | --- |
| IL1-α | –0.542 (0.121) | 0.522 (0.162) | 23.918 | 1 | <0.001 |
| IL1-β | –0.516 (0.124) | 0.510 (0.192) | 16.703 | 1 | <0.001 |
| IL1-RA | –0.171 (0.140) | 0.155 (0.176) | 2.011 | 1 | 0.173 |
| IL-2 | –0.575 (0.108) | 0.600 (0.188) | 26.407 | 1 | <0.001 |
| IL-2R | –0.525 (0.096) | 0.562 (0.200) | 21.710 | 1 | <0.001 |
| IL-3 | -0.532 (0.113) | 0.513 (0.146) | 27.817 | 1 | <0.001 |
| IL-4 | -0.509 (0.122) | 0.543 (0.152) | 25.993 | 1 | <0.001 |
| IL-5 | –0.470 (0.102) | 0.548 (0.142) | 31.676 | 1 | <0.001 |
| IL-6 | –0.630 (0.086) | 0.687 (0.134) | 61.720 | 1 | <0.001 |
| IL-7 | –0.332 (0.107) | 0.227 (0.122) | 10.440 | 1 | <0.001 |
| IL-8 | –0.495 (0.168) | 0.605 (0.169) | 22.194 | 1 | <0.001 |
| IL-9 | –0.471 (0.108) | 0.497 (0.177) | 19.648 | 1 | <0.001 |
| IL-10 | –0.475 (0.171) | 0.346 (0.172) | 11.371 | 1 | <0.001 |
| IL-12-P40 | –0.350 (0.102) | 0.332 (0.240) | 6.257 | 1 | 0.015 |
| IL-12-P70 | –0.530 (0.099) | 0.668 (0.276) | 16.801 | 1 | <0.001 |
| IL-13 | –0.370 (0.108) | 0.410 (0.315) | 4.923 | 1 | 0.031 |
| RIL-13 | –0.490 (0.129) | 0.408 (0.178) | 16.149 | 1 | <0.001 |
| IL-15 | –0.463 (0.093) | 0.513 (0.151) | 29.295 | 1 | <0.001 |
| IL-16 | –0.373 (0.069) | 0.373 (0.194) | 13.322 | 1 | <0.001 |
| IL-17 | –0.380 (0.153) | 0.410 (0.158) | 11.178 | 1 | <0.001 |
| IL-18 | –0.412 (0.117) | 0.465 (0.179) | 14.947 | 1 | <0.001 |
| TNF-α | –0.641 (0.076) | 0.783 (0.225) | 36.644 | 1 | <0.001 |
| TNF-β | –0.586 (0.089) | 0.733 (0.232) | 29.365 | 1 | <0.001 |
| TRAIL | –0.620 (0.088) | 0.677 (0.219) | 33.850 | 1 | <0.001 |

**IL:** interleukin; **IL-1RA:** interleukin-1 receptor antagonist; **TNF:** tumor necrosis factor; **TRAIL:** TNF-related apoptosis-inducing ligand.

**Supplementary Table 6**. **Differences in chemokines and growth factors between major depressive disorder (MDD) and healthy controls (HC).**

| **Variables (z scores)** | **HC (n=72)** | **MDD (n=72)** | **Wald Chi-square** | **df** | **p-value** |
| --- | --- | --- | --- | --- | --- |
| CCL2 (MCP-1) | –0.048 (0.140) | 0.109 (0.154) | 0.585 | 1 | 0.459 |
| CCL3 (MIP-1α) | –0.292 (0.227) | 0.454 (0.094) | 9.367 | 1 | 0.002 |
| CCL4 (MIP-1β) | –0.673 (0.104) | 0.687 (0.198) | 38.449 | 1 | <0.001 |
| CCL5 (RANTES) | –0.269 (0.222) | 0.079 (0.175) | 1.582 | 1 | 0.222 |
| CCL7 (MCP-3) | 0.026 (0.196) | 0.041 (0.173) | 0.004 | 1 | 0.949 |
| CCL11 (Eotaxin) | –0.141 (0.149) | 0.254 (0.172) | 3.326 | 1 | 0.077 |
| CCL27 (CTACK) | –0.394 (0.073) | 0.343 (0.090) | 36.644 | 1 | <0.001 |
| CXCL1 (GRO-α) | –0.510 (0.088) | 0.473 (0.162) | 25.657 | 1 | <0.001 |
| SDF1 | –0.587 (0.109) | 0.744 (0.220) | 28.596 | 1 | <0.001 |
| FGF | –0.506 (0.124) | 0.538 (0.150) | 25.504 | 1 | <0.001 |
| PDGF-B | –0.355 (0.112) | 0.264 (0.203) | 6.265 | 1 | 0.015 |
| VEGF | –0.228 (0.154) | 0.568 (0.203) | 11.597 | 1 | <0.001 |
| GCSF | –0.348 (0.166) | 0.252 (0.184) | 5.404 | 1 | 0.024 |
| GMCSF | –0.527 (0.097) | 0.618 (0.169) | 30.691 | 1 | <0.001 |
| HGF | –0.452 (0.109) | 0.410 (0.205) | 12.535 | 1 | <0.001 |
| IFN-α | –0.476 (0.114) | 0.493 (0.134) | 26.780 | 1 | <0.001 |
| IFN-γ | –0.458 (0.105) | 0.485 (0.253) | 10.767 | 1 | <0.001 |
| NGF | –0.499 (0.093) | 0.554 (0.139) | 35.423 | 1 | <0.001 |
| MIF | –0.405 (0.082) | 0.444 (0.154) | 21.922 | 1 | <0.001 |
| CXCL9 (MIG) | –0.287 (0.182) | 0.294 (0.210) | 3.762 | 1 | 0.060 |
| CXCL10 (IP-10) | –0.049 (0.160) | 0.164 (0.160) | 0.873 | 1 | 0.367 |
| SCF | –0.589 (0.103) | 0.723 (0.225) | 30.210 | 1 | <0.001 |
| SCGF | –0.377 (0.115) | 0.433 (0.202) | 12.963 | 1 | <0.001 |
| LIF | –0.444 (0.090) | 0.478 (0.120) | 33.120 | 1 | <0.001 |
| MCSF | –0.558 (0.097) | 0.594 (0.178) | 29.721 | 1 | <0.001 |

**CC:** C-C motif chemokine ligand; **CXCL:** C-X-C motif chemokine ligand; **SDF-1:** stromal cell-derived factor 1; **FGF:** fibroblast growth factor; **PDGF-B:** platelet-derived growth factor subunit B; **VEGF:** vascular endothelial growth factor; **G-CSF:** granulocyte colony-stimulating factor; **GM-CSF:** granulocyte-macrophage colony-stimulating factor; **HGF:** hepatocyte growth factor; **IFN:** interferon.
